# *In-silico* Investigation of Tubulin Binding Modes of a Series of Novel Antiproliferative Spiroisoxazoline Compounds Using Docking Studies

**Published:** 2015

**Authors:** Hoda Abolhasani, Afshin Zarghi, Maryam Hamzeh-Mivehroud, Ali Akbar Alizadeh, Javid Shahbazi Mojarrad, Siavoush Dastmalchi

**Affiliations:** a *Biotechnology Research Center, Tabriz University of Medical Sciences, Tabriz, Iran.*; b *Department of Medicinal Chemistry, School of Pharmacy, Tabriz University of Medical Sciences, Tabriz, Iran. *; c *Department of Medicinal Chemistry, School of Pharmacy, Shahid Beheshti University of Medical Sciences, Tehran, Iran. *

**Keywords:** Spiroisoxazoline, Molecular docking, Ligand protein interactions

## Abstract

Interference with microtubule polymerization results in cell cycle arrest leading to cell death. Colchicine is a well-known microtubule polymerization inhibitor which does so by binding to a specific site on tubulin. A set of 3', 4'-bis (substituted phenyl)-4'H-spiro [indene-2, 5'-isoxazol]-1(3H)-one derivatives with known antiproliferative activities were evaluated for their tubulin binding modes. 3D structures of the derivatives were docked into the colchicine binding site of tubulin using GOLD 5.0 program under flexible ligand and semi-flexible receptor condition. The spiroisoxazoline derivatives bind tubulin in a similar manner to colchicine by establishing at least a hydrogen bonding to Cys^241 ^as well as hydrophobic interactions with Leu^255^, Ile^378^ and Lys^254^ and few other residues at the binding pocket. It can be concluded that the spiroisoxazoline core structure common to the studied derivatives is a suitable scaffold for placing the antitubulin pharmacophoric groups in appropriate spatial positions required for tubulin binding activity.

## Introduction

**Figure 1:**
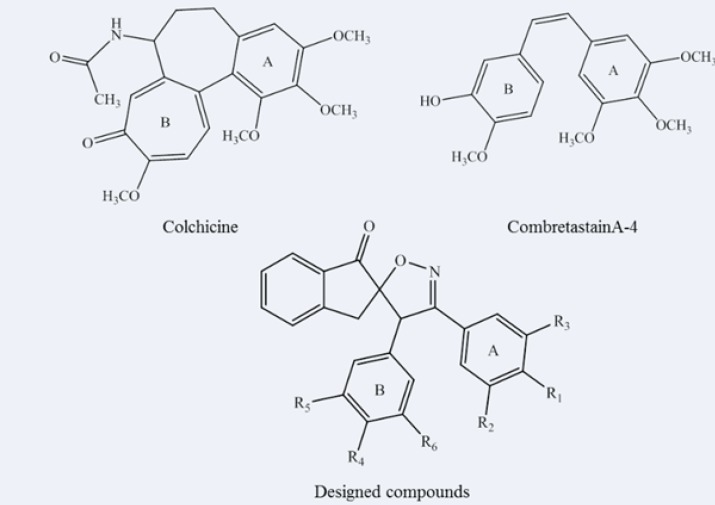
Chemical structures of antitubulin agents (Colchicine, CombretastainA-4) and our designed scaffold.

Cancer, one of the most fatal diseases, causes significant morbidity and mortality worldwide. Therefore, development of novel drugs to treat cancer is always needed (1). Inhibition of microtubule function causes serious cellular dysfunction leading to cell death. There are a number of small molecules, which bind tubulins at the colchicine binding site and interfere with microtubule polymerization, and hence arrest the cell cycle, leading to cell death (2-5). Numerous structure–activity relationship (SAR) studies have focused on features that bind to tubulin at the colchicine binding site, suggesting that the important pharmacophoric components include adjacent trimethoxyphenyl and *p*-methoxyphenyl units positioned in a *cis*-configuration as presented in Figure 1 (6-8). Although the improved potencies were found using the substituted monocycle-bridged analogues (9), however, compounds with the bicyclic systems as the bridge have also shown strong bioactivities (10). Based on the available SAR information, we have previously synthesized a new series of spiroisoxazolines and evaluated their antiproliferative activities (11). The aim of the current study was to show the key interactions of these novel molecules containing the spiroisoxazoline scaffold with tubulin as their potential target. These spiroisoxazoline derivatives can be considered as hybrid structures exhibiting aspects of combretastatin A-4 (Figure 1) as an inhibitor of the tubulin polymerization by binding at the colchicine site, and spiroisoxazoline structure as known anticancer scaffold (12-15).* In-vitro *studies also proposed cytotoxic effects for spiroisoxazoline type compounds on U937 lymphoma cells (16). Moreover, Bennani and co-workers have recently investigated the anti-breast cancer activity of some spiroisoxazoline derivatives (12, 15, 17). In this work, we report the docking studies on five spiroisoxazoline derivatives previously shown to have antiproliferative activities (Table 1) at the colchicine binding site. 

**Table 1:** Structure of 3',4'-bis (substituted phenyl)-4'H-spiro[indene-2,5'-isoxazol]-1(3H)-one derivatives 1-5.

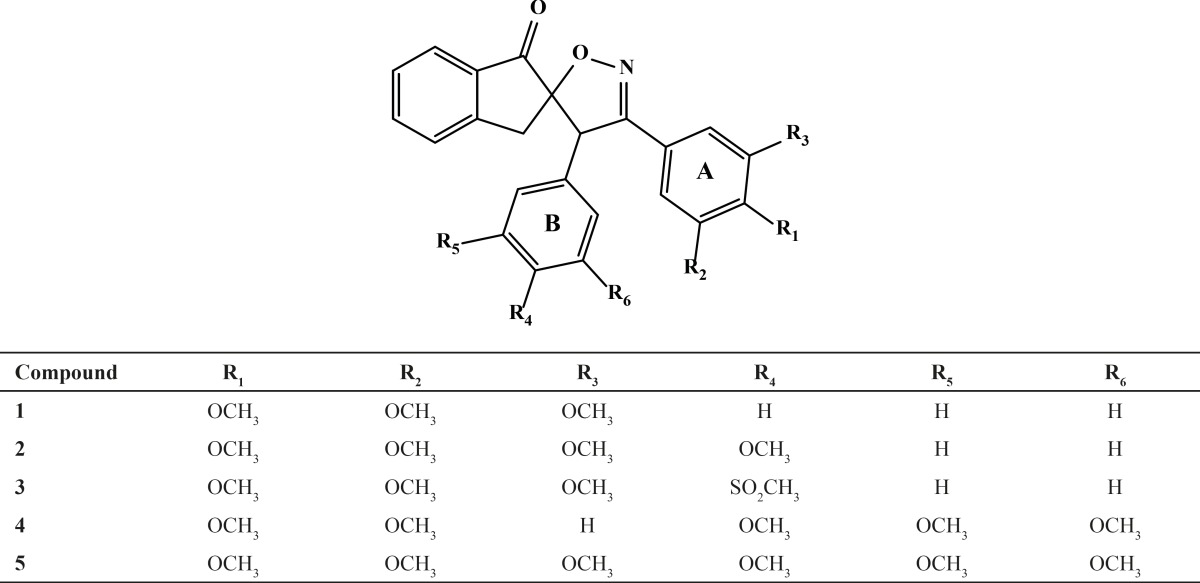

## Experimental

**Preparation of the compounds for docking study**

3D structures of the spiroisoxazoline derivatives were generated using HyperChem (version 7.0). The initial structures were first minimized using the MM+ force field (18). Then, those structures were fully optimized based on the semi-empirical quantum mechanics AM1 method, available in HyperChem (19). The output structures were converted to SYBYL Cartesian coordinate files (mol2 file format) using OpenBabel (version 2.3.2) in order to be imported into GOLD (Genetic Optimisation for Ligand Docking) for docking calculations (20, 21). The docking results were analyzed using PyMol software.

**Molecular docking study**

Application of *in-silico* methods such as quantitative structure-activity relationship (22), docking (23) and molecular modeling (24, 25) studies is one of the inevitable parts of modern drug design and development process. Flexible docking of the spiroisoxazoline derivatives (1**-**5) was carried out using the GOLD (version 5.0) program running under Linux OS. The crystal structure of tubulin (PDB code: 1SA0) was obtained from the Protein Data Bank at RCSB (http://www.rcsb.org). The binding cavity was determined based on the binding location of colchicine co-crystallized with tubulin, and then the colchicine molecule was removed and the spiroisoxazolines as well as colchicine itself were docked into the binding site. The protein structure was prepared for docking using GOLD, and docking was performed by defining a “point” central to the important residues involved in the binding. All atoms within a 10Å radius from the identified “point” were selected as the active atoms included in the calculations and then the flexible docking was carried out using force field parameters implemented into ChemPLP scoring function of GOLD suite. The interactions between ligands and tubulin at the colchicine binding site were visualized by using PyMOL (v0.99) program. 

## Results and Discussion

**Figure 2:**
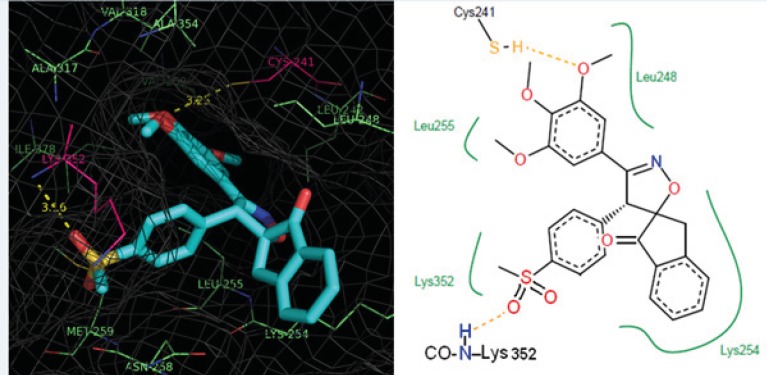
Representation of the binding mode of compound 3 (colored according to the atom type) at the colchicine binding site of tubulin illustrated using PyMOL v0.99. Tubulin experimental structure (PDB code 1SA0) was used for the docking calculation and the essential amino acid residues at the binding site are indicated. The yellow dotted lines show the hydrogen bonds and distances.

**Figure 3:**
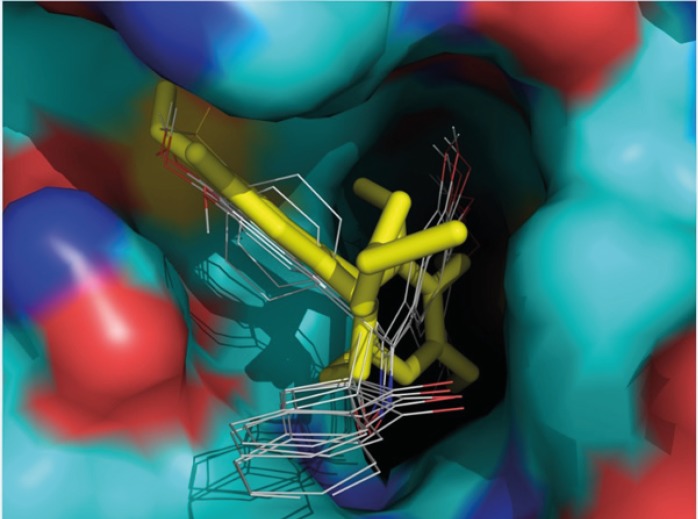
Three-dimensional superimposed representations of the colchicine (yellow and stick) and spiroisoxazoline compounds (1-5) (colored according to the atom type) docked at the colchicine binding site of tubulin using GOLD. 3D images were generated using PyMOL v0.99.

Five spiroisoxazoline derivatives containing antitubulin pharmacophoric elements were docked into the colchicines binding site of tubulin structure. Docking simulations were performed to predict the modes of interactions of the spiroisoxazoline compounds (1-5) with their tentative binding site. The ChemPLP scoring function uses the piecewise linear potential (f_PLP_) to model the steric complementarity between protein and ligand, in addition to the distance and angle dependent hydrogen and metal bonding terms (f_chem-hb_, f_chemcho_, f_chem-met_). The internal score of the ligand consists of the heavy-atom clash potential (f_lig-clash_) as well as the torsional potential (f_lig-tors_). ChemPLP fitness function is also capable of covalent docking (f_chem-cov_), considering flexible sidechains (f_chem-prot_) and explicit water molecules as well as handling constraints (f_cons_). The structural models of the compounds (1-5) bound to tubulin at the colchicine site are shown in Figures 2 and 3. Analysis of the docked pose of compound 1 in the colchicine binding site of tubulin demonstrates that it is stabilized by a hydrogen bond formed between the oxygen atom of the methoxy group in one of the *meta* positions on ring A and the sulfhydryl group of the Cys^241^ side chain (angle O---H—S =153.8°, distance = 2.1 Å). Whenever the *meta*-methoxy group on ring A is involved in hydrogen bonding, that group is positioned distal relative to the ring B. The patterns of hydrogen bonds formed between docked compounds and the binding site of tubulin are summarized in Table 2. 

**Table 2:** Hydrogen bonding patterns of spiroisoxazoline compounds 1-5 revealed from docking calculations.

**Compound**	**Hydrogen bonding pattern**			
	**Involved residue**	**Group form the compounds**	**Heavy atoms distances (Å)**	**Type (number) of H-bonds**
1	SH of Cys^241^	O atom of ring A *meta*-OMe	3.3	Two-center (1)
2	SH of Cys^241^	O atom of ring A *meta*-OMe O atom of ring A *para*-OMe	3.3 3.3	Three-center (1)
3	SH of Cys^241^ Amide H of Lys^352^	O atom of ring A *para*-OMe O atom of ring B para-SO2Me	3.3 3.2	Two-center (2)
4	SH of Cys^241^ H ND2 of Asn^258^	O atom of ring A *para*-OMe O atom of ring B *para*-OMe	3.4 3.1	Two-center (2)
5	SH of Cys^241^ H ND2 of Asn^258^	O atom of ring A *meta*-OMe O atom of ring B *para*-OMe	3.3 3.0	Two-center (2)

Furthermore, the 3,4,5- trimethoxy moieties on ring A of compound 1 enter deep into a hydrophobic pocket and interacts with hydrophobic residues Val^238^, Cys^241^, Leu^242^, Leu^255^, Ala^316^, Ala^317^, Val^318^ and Ile^378^. The phenyl moiety on 4’ position (ring B) and spiroisoxazolin motif occupy the remainder of the pocket and shows hydrophobic interactions with the side chains of Leu^248^, Ala^250^, Lys^254^, Asn^258^, Met^259^, and Lys^352^. The docked pose of compound 2 at the colchicine binding site of tubulin demonstrates an interaction pattern very similar to that of compound 1 outlined above with the 3,4,5-trimethoxy groups on phenyl ring A involved in hydrophobic interactions with the side chains of Leu^242^ and leu^255^. The 4-methoxy phenyl (ring B) at the 4’ position of this compound interacts with residues Lys^352^ and Met^259^. One noticeable difference is that the binding of compound 2 is further stabilized by a three-center hydrogen bond involving the SH of Cys^241^ and two oxygens of methoxy groups on ring **A** at the *meta* (angle O_o_---H—S =130.1°, distance = 2.3 Å) and *para* (angle O_p_---H—S =124.0°, distance = 2.4 Å) positions. Compound 3 when docked into the colchicine binding site of tubulin (Figures 2) shows two hydrogen bonds, one between the *para*-methoxy group of ring A with Cys^241^ (angle O_p_---H—S =125.5°, distance = 2.3 Å), and the other between the oxygen atom of the sulfonyl (>SO2) functional group of 4-methylsulfonyl moiety on ring B with the amide hydrogen of Lys^352^ (angle O---H—N =124.2°, distance = 2.5 Å) residue. In addition, the 3,4,5- trimethoxyphenyl moiety at 3’ position of compound 3 enters the hydrophobic pocket and interacts with Val^238^, Leu^242^, Ala^316^, Ala^317^, Val^318^ and Ile^378^. The 4-methylsulfonyl group on ring B also makes hydrophobic contacts with the apolar residues in the binding pocket described previously. In compounds 4 and 5 it is clear that the hydrophobic pocket of the colchicine binding site was occupied by rings A and B plus the groups substituted on these rings. Additionally, the binding of compounds 4 and 5 to tubulin is stabilized through two hydrogen bonds. In the case of former compound, one H-bond is formed between the ring A *para*-methoxy group and side chain of Cys^241 ^and the other is formed between ring B *para*-methoxy group and one of the hydrogens of ND2 nitrogen of Asn^258^. These H-bond interactions are also seen for compound 5 with the difference that instead of the *para*, it is the *meta*-methoxy oxygen which is involved in H-bonding with Cys^241^. The residues noted were also found to be involved in the binding of colchicine with tubulin. Figure 3 shows three-dimensional superimposition of colchicine and compounds 1-5 while docked into the colchicine binding site and supports the idea that the active compounds are well incorporated in the binding pocket.

## Conclusion

Introducing novel and efficient approaches to treat cancer has been a goal for many projects, and the development of new antitumor agents plays an important role toward fulfilling this aim. Investigation of the hydrogen bonding patterns revealed from the docking of the studied antiproliferative spiroisoxazolines shows that both of the methoxy groups located at the *para* and distal-*meta* positions of ring A of these compounds are capable of providing the acceptor oxygen atoms in the hydrogen bonding interactions to Cys241. This study indicates that 3',4'-bis (substituted phenyl)-4'H-spiro[indene-2,5'-isoxazol]-1(3H)-one is a suitable scaffold for designing new antitubulin agents, such as those derivatives presented in this study, and may be helpful in the search for novel classes of potent anticancer agents. In summary, the results of the molecular docking studies indicate the importance of the spiroisoxazolines skeleton and 3,4,5-trimethoxyphenyl moiety in establishing hydrogen bonds and hydrophobic interactions in a synergistic fashion leading to proper tubulin binding capabilities of compounds possessing such groups. Accordingly, the results may suggest that the studied compounds interact with tubulin in a similar fashion observed for colchicines, and hence strongly recommend biological evaluation of the designed compounds for their anti-tubulin activities using specific tests 

## References

[1] Mukherjee AK, Basu S, Sarkar N, Ghosh AC (2001). Advances in cancer therapy with plant based natural products. Curr. Med. Chem.

[2] Chinigo GM, Paige M, Grindrod S, Hamel E, Dakshanamurthy S, Chruszcz M, Minor W, Brown ML (2008). Asymmetric synthesis of 2,3-dihydro-2-arylquinazolin-4-ones: methodology and application to a potent fluorescent tubulin inhibitor with anticancer activity. J. Med. Chem.

[3] Hamel E (1996). Antimitotic natural products and their interactions with tubulin. Med. Res. Rev.

[4] Kim DY, Kim KH, Kim ND, Lee KY, Han CK, Yoon JH, Moon SK, Lee SS, Seong BL (2006). Design and biological evaluation of novel tubulin inhibitors as antimitotic agents using a pharmacophore binding model with tubulin. J. Med. Chem.

[5] Romagnoli R, Baraldi PG, Carrion MD, Cruz-Lopez O, Cara CL, Basso G, Viola G, Khedr M, Balzarini J, Mahboobi S, Sellmer A, Brancale A, Hamel E (2009). 2-Arylamino-4-amino-5-aroylthiazoles One-pot synthesis and biological evaluation of a new class of inhibitors of tubulin polymerization.. J. Med. Chem.

[6] Hsieh HP, Liou JP, Mahindroo N (2005). Pharmaceutical design of antimitotic agents based on combretastatins. Curr. Pharm. Des.

[7] Pinney KG, Jelinek C, Edvardsen K, Chaplin DJ, Pettit GR, Cragg GM, Kingston DGI, Newman DJ (2005). The discovery and development of combretastatins. Anticancer agents from natural products.

[8] Tron GC, Pirali T, Sorba G, Pagliai F, Busacca S, Genazzani AA (2006). Medicinal chemistry of combretastatin A4: present and future directions. J. Med. Chem.

[9] Sanchez Maya AB, Perez-Melero C, Salvador N, Pelaez R, Caballero E, Medarde M (2005). New naphthylcombretastatins. Modifications on the ethylene bridge. Bioorg. Med. Chem.

[10] Flynn BL, Hamel E, Jung MK (2002). One-pot synthesis of benzo[b]furan and indole inhibitors of tubulin polymerization. J. Med. Chem.

[11] Abolhasani H, Zarghi A, Abolhasani A, Hamzeh-Mivehroud M, Bargahi N, Notash B, Shahbazi Mojarrad J, Dastmalchi S (2014). Design, synthesis and in vitro cytotoxicity evaluation of new 3',4'-bis (3,4,5-trisubstituted)-4'H-spiro[indene-2,5'-isoxazol]-1(3H)-one derivatives as promising anticancer agents. Lett. Drug Des. Discov.

[12] Howe RK, Shelton BR (1990). Spiroheterocycles from the reaction of nitrile oxides with 3-methylenephthalimidines. J. Org. Chem.

[13] Khazir J, Singh PP, Reddy DM, Hyder I, Shafi S, Sawant SD, Chashoo G, Mahajan A, Alam MS, Saxena AK, Arvinda S, Gupta BD, Kumar HM (2013). Synthesis and anticancer activity of novel spiro-isoxazoline and spiro-isoxazolidine derivatives of alpha-santonin. Eur. J. Med. Chem.

[14] Shin KD, Lee MY, Shin DS, Lee S, Son KH, Koh S, Paik YK, Kwon BM, Han DC (2005). Blocking tumor cell migration and invasion with biphenyl isoxazole derivative KRIBB3, a synthetic molecule that inhibits Hsp27 phosphorylation. J. Biol. Chem.

[15] Smietana M, Gouverneur V, Mioskowski C (1999). A new access to spiro-isozazolines derivatives. Tetrahedron Lett.

[16] Najim N, Bathich Y, Zain MM, Hamzah AS, Shaameri Z (2010). Evaluation of the bioactivity of novel spiroisoxazoline typecompounds against normal and cancer cell lines. Molecules.

[17] Bennani B, Kerbal A, Ben Larbi N, Ben Hadda T (2004). Synthesis and application of isothiochromeno[3,4-e][1,2]oxazine (TCO) as new antitumoral agents. OMPIC.

[18] Allinger NL (1977). Conformational analysis 130 MM2. A hydrocarbon force field utilizing v1 and v2 torsional terms.. J. Am. Chem. Soc.

[19] Dewar MJS, Thiel W (1977). Grond states of molecules. MNDO results for molecules containing hydrogen, carbon, nitrogen and oxygen. J. Am. Chem. Soc..

[20] Jones G, Willett P, Glen RC (1995). Molecular recognition of receptor sites using a genetic algorithm with a description of desolvation. J. Mol. Biol.

[21] Morris GM, Goodsell DS, Halliday RS, Huey R, Hart WE, Belew RK, Olson AJ (1998). Automated docking using a Lamarckian genetic algorithm and empirical binding free energy function. J. Comput. Chem.

[22] Dastmalchi S, Hamzeh-Mivehroud M, Asadpour-Zeynali K (2012). Comparison of different 2D and 3D-QSAR methods on activity prediction of histamine H3 receptor antagonists. Iran. J. Pharm. Res.

[23] Dastmalchi S, Hamzeh-Mivehrod M (2005). Molecular modelling of human aldehyde oxidase and identification of the key interactions in the enzyme-substrate complex. Daru.

[24] Dai Y, Chen N, Wang Q, Zheng H, Zhang X, Jia S, Dong L, Feng D (2012). Docking analysis and multidimensional hybrid QSAR model of 1,4-Benzodiazepine-2,5-Diones as HDM2 antagonists. Iran. J. Pharm. Res.

[25] Dastmalchi S, Church BW, Morris MB (2008). Modelling the structures of G protein-coupled receptors aided by three-dimensional validation. BMC Bioinform.

